# High adsorption rate is detrimental to bacteriophage fitness in a biofilm-like environment

**DOI:** 10.1186/1471-2148-9-241

**Published:** 2009-10-05

**Authors:** Romain Gallet, Yongping Shao, Ing-Nang Wang

**Affiliations:** 1 Department of Biological Sciences, University at Albany, Albany, NY 12222, USA; 2 Current address : CEFE - UMR 5175, 1919 route de Mende, F-34293 Montpellier cedex 5, France; 3 Current address : Thomas Jefferson University, Kimmel Cancer Center, Department of Cancer Biology, 233 S. 10th Street, BLSB Room 522, Philadelphia, PA 19107, USA

## Abstract

**Background:**

Bacterial biofilm is ubiquitous in nature. However, it is not clear how this crowded habitat would impact the evolution of bacteriophage (phage) life history traits. In this study, we constructed isogenic λ phage strains that only differed in their adsorption rates, because of the presence/absence of extra side tail fibers or improved tail fiber J, and maker states. The high cell density and viscosity of the biofilm environment was approximated by the standard double-layer agar plate. The phage infection cycle in the biofilm environment was decomposed into three stages: settlement on to the biofilm surface, production of phage progeny inside the biofilm, and emigration of phage progeny out of the current focus of infection.

**Results:**

We found that in all cases high adsorption rate is beneficial for phage settlement, but detrimental to phage production (in terms of plaque size and productivity) and emigration out of the current plaque. Overall, the advantage of high adsorption accrued during settlement is more than offset by the disadvantages experienced during the production and emigration stages. The advantage of low adsorption rate was further demonstrated by the rapid emergence of low-adsorption mutant from a high-adsorption phage strain with the side tail fibers. DNA sequencing showed that 19 out of the 21 independent mutant clones have mutations in the *stf *gene, with the majority of them being single-nucleotide insertion/deletion mutations occurring in regions with homonucleotide runs.

**Conclusion:**

We conclude that high mutation rate of the *stf *gene would ensure the existence of side tail fiber polymorphism, thus contributing to rapid adaptation of the phage population between diametrically different habitats of benthic biofilm and planktonic liquid culture. Such adaptability would also help to explain the maintenance of the *stf *gene in phage λ's genome.

## Background

The bacterial biofilm is a dynamic, surface-associated structure often consisted of many different species of bacterial populations embedded in an exopolysaccharide matrix secreted by the bacteria. For many bacterial species, existence in the benthic habitat of the biofilm is an integral part of their life cycles [1,2]. The bacterial biofilm is initiated by the settlement (attachment) of planktonic bacteria in the environment onto a surface, forming microcolonies. This is followed by subsequent growth and maturation of the biofilm structure. Dispersal of newly emerged bacterial cells from the existing biofilm structure helps to disseminate the formation of bacterial biofilms to new locations [3].

It is generally believed that biofilms structures are resistant to many stresses, including bacteriophage (phage) infections [2,4]. However, several studies have demonstrated that phages can indeed infect biofilms in an experimental setting [5-7], suggesting that this phenomenon probably occurs in nature as well. The life cycle of a biofilm-infecting phage can be hypothesized to be consisted of three stages: (1) settlement, or attachment, of phages onto bacterial cells embedded in the biofilm matrix, (2) production of phage progeny inside the biofilm matrix, and (3) emigration, or diffusion, of the newly emerged phages out from the current focus of infection into liquid carrier for translocation to new biofilm patches. From the phage's point-of-view, the most important difference between the planktonic, free-floating and the benthic, biofilm-bound environments would be the number of bacterial cells it may potentially encounter in its immediate vicinity. Depending on the bacterial species, the available nutrient, and the underlying substratum, the cell densities in a biofilm have been shown to range from 10^5 ^to 10^8 ^cfu/cm^2^, with thickness at the tens to hundreds of μm range [8-10]. By converting the above definition of cell density to that based on the volumetric unit, the cell density in the biofilm would in most cases be in excess of 10^9 ^cfu/mL, a much higher concentration than commonly encountered in the planktonic state (*e.g*., liquid culture). In fact, one characteristic of the biofilm is the high cell density [4]. Therefore, it is not simply a speculation that the phages would often encounter a much more concentrated host population when inside the biofilm.

For the few studies focusing on the effect of host density on the evolution of phage life history traits [11,12], the general conclusion is that a higher cell density in the environment would select for phages with a shorter lysis time (latent period), and vice versa. Though not directly related to the effect of host density, an empirical study also showed that the phage adsorption rate is important in determining the competition outcome [13]. All else being equal, the phage with a higher adsorption rate would have a higher fitness as well. Since all these studies assumed or used the planktonic state of the bacterial cells (*i.e*., the homogeneously mixed liquid culture), it is not clear if these general conclusions can also be extended to the condition with densely populated bacterial cells embedded in the biofilm matrix. However, we can imagine that the success of a phage in a biofilm condition would probably depend on its ability to competitively complete the aforementioned stages of settlement-production-emigration.

In this study, we used the agar-entrapped bacterial cells and the agar matrix as a simulacrum [14] for the much more intricate biofilm structure [2] to explore the effect of a biofilm-like environment on the evolution of a phage life history trait, specifically the adsorption rate. We constructed three isogenic strains of λ phage, each with a different adsorption rate, and determined their abilities in completing the settlement-production-emigration cycle. We found that high adsorption rate is beneficial to settlement, but detrimental to production and emigration. Overall, our results showed that low adsorption rate is more advantageous under the biofilm-like environment. Furthermore, we also witnessed strong selection of low adsorption rate phages in our experimental setup.

## Results

### Construction of isogenic λ phages with different adsorption rates and marker states

**Figure 1:**
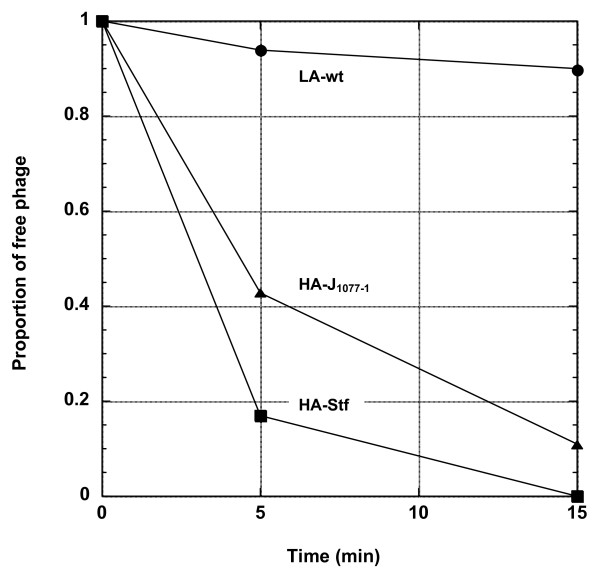
**Examples of adsorption profiles of HA-Stf, HA-J1077-1, and LA-wt phages**. The proportion of free phages remaining in solution is plotted against time. Symbols: circle, LA-wt; square, HA-Stf; triangle, HA-J1077-1. It is to be noted that for LA-wt and HA-J1077-1 phages ~108 cells/mL of stationary E. coli XL1 Blue cells were used, while ~107 were used for HA-Stf.

To assess the impact of phage adsorption rate on the settlement, production, and emigration stages of the phage life cycle in a biofilm-like environment, *e.g*., the top agar gel, two types of high-adsorption (HA) λ strains were constructed: (1) the HA-Stf (with the genotype of *stf*^+^), which carries the side tail fibers, and (2) the HA-J_1077-1 _(with the genotype *J*_1077-1_), a host range mutant carrying a modified tail fiber protein J [15]. As shown in Figure 1, when compared to the parental wild-type low-adsorption (LA-wt) laboratory λ strain, from which both HA strains were derived, these two HA strains showed markedly increased ability in adsorbing onto the bacteria host cells. The estimated adsorption rates are 1.03 ± 0.23 × 10^-8^, 1.85 ± 0.63 × 10^-9^, and 7.44 ± 5.47 × 10^-11 ^phage^-1^cell^-1^mL^-1^min^-1 ^for HA-Stf, HA-J_1077-1_, and LA-wt, respectively. However, it is to be noted that these adsorption rates were estimated using cells in stationary phase, not the customary exponential phase, and without the addition of Mg^2+^. It has been shown that phages adsorb much less readily under the condition used in this study [16]. Nevertheless, even with the current assay condition, the ranking of adsorption rates among these phages is HA-Stf > HA-J_1077-1 _>> LA-wt.

Two isogenic markers (*lacZα*^+^, generating blue plaque, and *lacZα*^-^, generating clear "white" plaque) were also engineered into the genomes of the above phage strains by fusing the α-fragment, encoded by *E. coli*'s *lacZ *gene, with the phage's endolysin protein R (encoded by the *R *gene) [13]. In liquid culture, there is no discernible marker effect on phage fitness [13]. However, under the experimental conditions used in this study we observed that "white" plaques contain, on average, 16% more phages than the isogenic blue counterpart (χ^2^_7 _= 19.19, *p *= 0.0018). That is, under our assay condition, there is a slight, but significant cost associated with the blue marker. To minimize the marker effect, all the results described below were conducted with both marker states (see Materials and Methods section for details).

### Effect of adsorption rate on settlement rate

**Figure 2:**
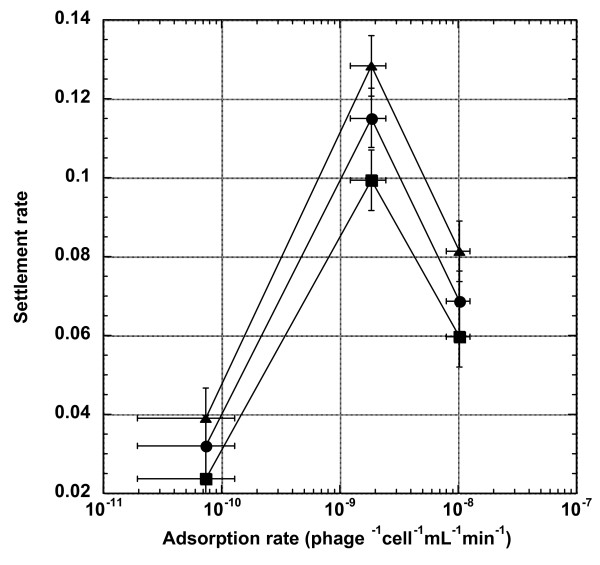
**Effect of adsorption rate on phage settlement rate**. The proportions of phage populations in the liquid medium that settled on the surface (the settlement rate) of different top agar gel concentrations were plotted against adsorption rates. Symbols: triangles, 0.27% top agar gel concentration; circles, 0.53%; squares, 0.8%. Error bars represent 95% confidence intervals.

In a well-mixed liquid culture, all else being equal, the phage with the higher adsorption rate would, on average, have a shorter time adsorbing onto a susceptible host, thus resulting in a shorter generation time and a higher growth rate (fitness) [12,13]. Therefore, when moving from the liquid environment to the biofilm-like environment, it is expected that the phage with a higher adsorption rate would also be able to settle (adsorb) more quickly onto the susceptible hosts embedded in the biofilm matrix. To test this hypothesis, we conducted settlement experiments by allowing phages present in the liquid medium to adsorb onto the bacterial cells embedded in three different concentrations of top-agar gel (0.27%, 0.53%, and 0.8%) for a prescribed period of time. As shown in Figure 2, both HA phages have higher fractions of phages settling onto the bacterial lawn. *Post hoc *unplanned comparisons among means using the Tukey-Kramer HSD test (as implemented in JMP version 7.0.2 statistical software) showed that, irrespective of the top-agar concentrations, the ranking of settlement rates is HA-J_1077-1 _> HA-Stf > LA-wt. As would be expected, the settlement rate was also influenced by the top-agar concentrations. Again, unplanned comparisons among means showed that the settlement rates are significantly different between 0.27% and 0.8%, but not between other comparisons.

Interestingly, our result seems to suggest the existence of an optimal adsorption rate for phage settlement. We found that the HA-J_1077-1 _strain, while having the intermediate adsorption rate, has the highest settlement rate. Nevertheless, we still observe the expected positive correlation between the adsorption rate and the settlement rate when comparing between the LA and HA phages. It is only by comparing between HA-Stf and HA-J_1077-1 _did we witness the contrary to our expectation. One possible explanation may lie in the somewhat different morphology of these phage strains. HA-Stf's extra side tail fibers, extending out from the phage virion, would make it bulkier than the other two strains, which are expected to have the same shape. The extra hydrodynamic volume, while inconsequential when in liquid culture, could impede HA-Stf's ability to efficiently diffuse into the biofilm matrix to gain access to the embedded cells. Overall, our results showed that, all else being equal, high adsorption rate would also result in high settlement rate. But the morphology of phage virion could also be expected to exert its effect in the biofilm-like environment.

### Higher adsorption rate results in smaller plaque size and lower productivity

Once the infection plaque was formed, two factors may affect the phage progeny's prospect of migrating out of the current plaque: (1) the size of the plaque and (2) the productivity of the plaque. All else being equal, we assume that a larger plaque size would provide a larger surface area for the phage progeny to migrate out of the current plaque. The same rationale also applies to plaque productivity.

We determined the plaque size for each phage strain. As the analysis showed, the phage strain has a significant effect on the plaque size (at 0.8% top-agar concentration, *F*_(2,23) _= 26.09, *p *< 0.0001). Furthermore, unplanned comparisons among means showed that the ranking of the plaque size is LA-wt (3.36 ± 0.360 mm^2^, mean ± 95% confidence interval) > HA-J_1077-1 _(2.02 ± 0.339 mm^2^) ≈ HA-Stf (1.73 ± 0.339 mm^2^).

**Figure 3:**
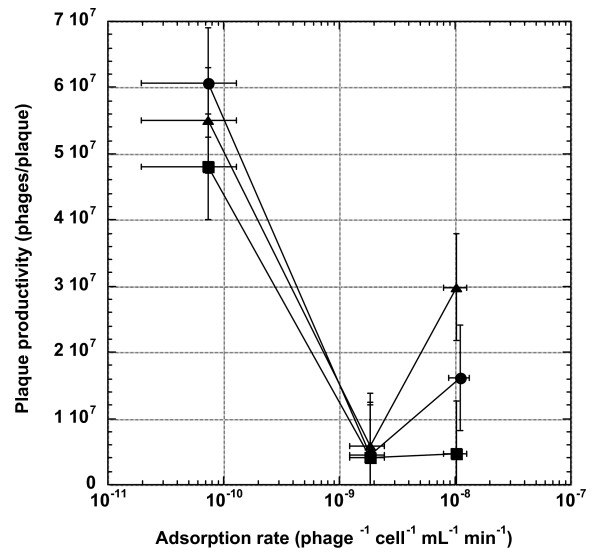
**Effect of adsorption rate on phage plaque productivity**. Numbers of phages per plaque were plotted against adsorption rates under different top agar concentrations. Symbols: triangles, 0.27% top agar gel concentration; circles, 0.53%; squares, 0.8%. Error bars represent 95% confidence intervals. The data point for HA-Stf phage with 0.53% top agar gel concentration was shifted slightly to the right to increase clarity of the graph.

We also determined the plaque productivity for each phage strain under different top-agar concentrations (Figure 3). Unplanned comparisons among means showed that the ranking of plaque productivity, irrespective of the top-agar concentrations, is LA-wt > HA-Stf ≈ HA-J_1077-1 _(except for when the top agar concentration is 0.27%, in which case the ranking is LA-wt > HA-Stf > HA-J_1077-1_). Interestingly, depending on whether the phage strain has side tail fibers or not, the top agar concentration has a differential effect on plaque productivity. For the LA-wt and the HA-J_1077-1 _strains (both without side tail fibers), the plaque productivity is not significantly affected by the top agar concentration, while for the HA-Stf strain it is negatively correlated with the top agar concentration (top agar concentration × strain interaction: χ^2^_7 _= 609.8, *p *< 0.0001). Again, unplanned comparisons among means showed that the HA-Stf productivities are significantly different between the 0.8% and 0.27% top agar concentrations (about 6.5-fold), but not between the other comparisons.

### Higher adsorption rate results in lower emigration rate

**Figure 4:**
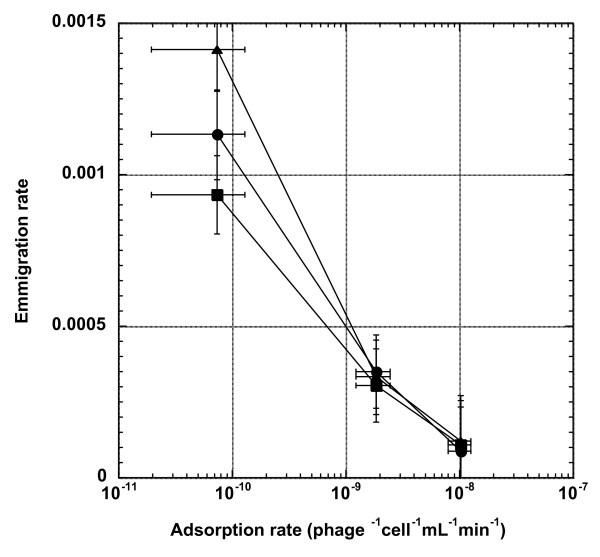
**Effect of adsorption rate on phage emigration rate**. The proportions of phage populations embedded inside the top agar gel that diffused out of the top agar gel (the emigration rate) were plotted against adsorption rates under different top agar gel concentrations. Symbols: triangles, 0.27% top agar gel concentration; circles, 0.53%; squares, 0.8%. Error bars represent 95% confidence intervals.

Once the progeny phages have emerged from the current plaque, they would need to diffuse out of the confine of the plaque in order to start new infections. We hypothesized that, all else being equal, more high-adsorption phages would be found associated with the host cells and cell debris, rather than being free-floating in the biofilm matrix, thus contributing to their reduced chance of diffusing out of the plaque. To test such a possibility, we determined the emigration rates of all three phage strains under different top agar concentrations. In this case, the emigration rate is defined as the proportion of plaque-embedded phage progeny that have successfully diffused out of the top agar gel within a prescribed 30 min period. The results are shown in Figure 4. Unplanned comparisons among means showed that, across all top agar concentrations, the LA-wt phage has the highest emigration rate, ranging from 0.09% to 0.14%, depending on the top agar concentrations. On the other hand, there is virtually no difference in emigration rates between the two HA phages (~0.01% for HA-Stf and ~0.03% for HA-J_1077-1_), except when the top agar concentration is 0.8%, in which case the HA-J_1077-1_'s emigration rate is significantly higher than that of the HA-Stf.

Because HA phage have higher settlement rates than the LA phage, it is possible that the observed low emigration rates for the HA phages is simply due to their higher probabilities of re-settlement during the emigration process. That is, all phage strains may have the same emigration rate, but the quicker re-settlement of the HA phages contributes to the observed lower emigration rates for the HA phages. Two observations indicate that settlement rate has minimal, if any, influence on the determination of the emigration rate. First, the differences in settlement rate among all phage strains across all top agar gel concentrations is three- to four-fold (mainly between HA-J_1077-1 _and LA-wt), while the differences in the determined emigration rate is between eight- to thirteen-fold (mainly between HA-Stf and LA-wt) (see Table 1). The smaller difference in the settlement rates can not completely account for the much larger difference in the determined emigration rates. Second, if the settlement rate has the dominant effect on the determined emigration rate, then HA-J_1077-1_, having the highest settlement rate, should also have the lowest emigration rate, but it is not the case.

**Table 1:** Effect of adsorption rate on the relative advantage of each stage of phage life cycle^*a*^

		**Stage of life cycle**											
					
		**Settlement**			**Production**			**Emigration**			**Cumulative effect**^*b*^		
Top agar concentration		0.27%	0.53%	0.80%	0.27%	0.53%	0.80%	0.27%	0.53%	0.80%	0.27%	0.53%	0.80%

Phage strains	LA-wt	1.00	1.00	1.00	1.00	1.00	1.00	1.00	1.00	1.00	1.00	1.00	1.00
	
	HA-J_1077-1_	3.29	3.60	4.18	0.11	0.07	0.08	0.24	0.31	0.33	0.09	0.08	0.11
	
	HA-Stf	2.10	2.15	2.50	0.54	0.26	0.10	0.09	0.08	0.12	0.10	0.04	0.03

^*a *^All values are relative to the those of the LA-wt strain. ^*b *^The cumulative effect is calculated as Settlement × Production × Emigration.

### Relative effect of adsorption rate on the settlement-production-emigration cycle

To gain further insight into the cumulative effect of adsorption rate on the entire settlement-production-emigration cycle, the above results from the three λ strains were converted into relative scales. Table 1 shows the relative effects the adsorption rate has on each stage of the settlement-production-emigration cycle. For example, for the condition of 0.8% top agar, the HA-J_1077-1 _phage would have 4.18-fold advantage over the LA-wt phage in settling on new hosts embedded in top agar. That is, for every LA-wt phage settlement, there would have 4.18 settlement for the HA-J_1077-1 _phage. However, for every LA-wt phage produced in the resulting plaque, only 0.08 HA-J_1077-1 _phage would be produced. Furthermore, for every LA-wt phage that eventually emigrated out of the current plaque, only 0.33 HA-J_1077-1 _phage was able to emigrate out. Cumulatively, for every LA-wt phage that successfully passed through the settlement-production-emigration cycle, only 0.11 HA-J_1077-1 _phage was able to complete the cycle. As shown in Table 1, high-adsorption phages are tremendously disadvantageous in the biofilm environment.

### Impact of adsorption rate on phage production-emigration in the biofilm environment

**Figure 5:**
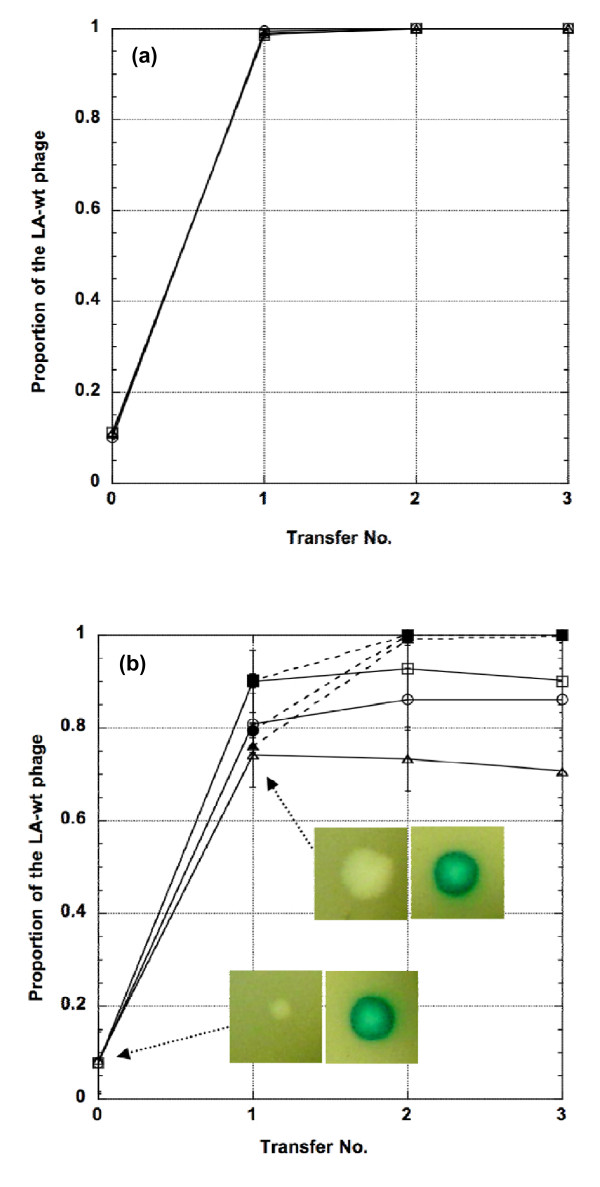
**Competitive serial transfer experiments between LA-wt and (a) HA-J_1077-1 _or (b) HA-Stf**. Curves represent the proportion of LA-wt in the carrier liquid medium as a function of transfer number. The experiments were performed at 0.27 (triangle), 0.53 (circle) and 0.8% (square) top agar concentrations. On (b), solid lines and filled symbols correspond to actual competitive transfer experiments, while dotted lines and open symbols correspond to reconstruction experiments (see Results). The insets show the of HA-Stf (white) and LA-wt (blue) plaques, before and after the first transfer. Error bars represent 95% confidence intervals.

The overwhelming advantage of the low-adsorption phages in the biofilm environment is demonstrated by the competitive production-emigration transfer experiments. We mixed roughly 10% of LA-wt and 90% of either HA-Stf or HA-J_1077-1 _and plated on the same agar plate. Since the phage strains are marked with either the LacZα^+ ^or lacZα^- ^fragment (see Materials and Methods), the phage strains are easily distinguishable by the chromogenic reaction on the agar plate. Progeny phages emerged from the resulting plaques were allowed to diffuse out of the plaques into the liquid medium to simulate the spread of emerging phages via carrier liquid phase. Figure 5 shows LA-wt phage's remarkable ability to increase its representation in the liquid phase. When compared to the HA-J_1077-1 _phage, the relative frequency of the LA-wt phage increased from the initial 10% to >98% after just a single production-emigration event. Interestingly, the LA-wt phage's advantage over HA-J_1077-1 _is similar across all three top agar concentrations (0.27%, 0.53%, and 0.8%; χ^2^_4 _= 1.52, *p *= 0.47 - Figure 5a). Similarly, when compared to the HA-Stf phage, the relative frequency of the LA-wt phage increased from the initial of 10% to 74-90% (depending on the top agar gel concentration) after just one production-emigration event. Furthermore, in this case, the top agar concentration has a much more pronounced effect on the phage's relative frequency. As would be expected, the higher the top agar concentration, the larger the impediment it has on the HA-Stf phage (χ^2^_4 _= 10.97, *p *= 0.0119) (Figure 5b).

### Selection of LA phages in biofilm-like environment

Even though the LA-wt phage has an enormous advantage in the production-emigration cycle when compared to either the HA-Stf or HA-J_1077-1 _phages, it is striking to observe that, even after several rounds of serial production-emigration experiments, the LA-wt phage's relative frequency was persistently maintained at 74-90% in the presence of HA-Stf (see Figure 5b), unlike the expected fixation when competing against the HA-J_1077-1 _phage (see Figure 5a). For all 36 replicate experiments of LA-wt vs. HA-Stf phages, none of the populations has experienced the fixation of the LA-wt phage. The inability of the LA-wt phage to be fixed in the presence of the HA-Stf phage is concomitant with the appearance of larger plaques bearing the marker for the HA-Stf phage. That is, instead of a mix of small and large plaques as in the initial population, the subsequent serially transferred populations contained only large plaques (see Figure 5b, insets). We hypothesized that the large-plaque variants of the HA-Stf phage (called GP, Grande Plage) may have become low-adsorption by losing its side tail fibers, thus rendered it genotypically and phenotypically the same as the LA-wt phage. As a consequence, only the side-tail-fiber-less phages were able to diffuse out of the top agar.

To test this hypothesis, we conducted reconstruction experiments using fresh HA-Stf phages from the original phage stock, rather than the phages that have diffused into the liquid medium after the production-emigration experiment, to reconstitute the proportion of the HA-Stf phage before conducting the next transfer. For example, if after the first production-emigration experiment the phage population contains 25% of the HA-Stf phage, then 25% of HA-Stf and 75% of LA-wt phages would be withdrawn from the original phage stocks to simulate the proportion before the next production-emigration process starts. Presumably, the reconstituted phage population would remove the effect of selection during the production-emigration process. As expected, under such an experimental condition, the frequency of the LA-wt phage was fixed in subsequent transfers (see Figure 5b, dotted lines).

**Figure 6:**
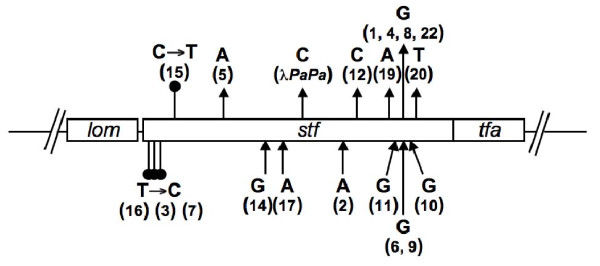
**Map of mutations in the GP phages**. Shown is a schematic representation of the section of the HA-Stf genome, containing the *lom*, *stf*, and the *tfa *genes. Vertical lines indicate the positions of all the mutations listed in [additional file 1], with the missense mutations shown as filled circles, deletion mutations as arrowheads pointing away from the bar, and insertion mutations as arrowheads pointing toward the bar. Also shown were the nucleotide changes. Numbers in the brackets showed the GP strain numbers [additional file 1]. Great majority of the laboratory λ strains have the λ*PaPa *mutation [35], including our LA-wt strain.

To find out the genetic basis for the observed large plaque morphology, 21 independent GP mutants were isolated and genes involved in tail fiber (only the last 20% of the *J *sequence), λ outer membrane (*lom*), side tail fiber (*stf*, originally *orf401 *and *orf314*), and tail fiber assembly (*tfa*) were sequenced. The results are shown in Figure 6 and [additional file 1]. As shown in Figure 6, 19 out the 21 isolates had a mutated *stf *gene. Curiously, no mutation was found in the sequenced region in the remaining two isolates. Out of these 19 isolates, 15 carry a frameshift and 4 missense mutations. Interestingly, all the missense mutations were found clustered at the 5' end of the gene while the frameshift mutations were distributed throughout the rest of the gene, but unevenly (Figure 6). More than half of the insertion/deletion mutations were clustered around the last 20% of the gene. In fact, four independent deletions were found at nucleotide position 21572 (based on GenBank accession No. NC_001416) and two insertions at the next position, 21573 (Supplemental Table two). Closer inspection of the gene sequences showed that, with one exception (GP20), all the insertion/deletion mutations occurred in regions with short homonucleotide runs in the sequence. It has been long known that, possibly due to slippage of DNA polymerase during DNA replication [17], homonucleotide stretches are hypermutable in generating insertion/deletion mutations [18-20]. Presumably all these mutations are loss-of-function mutations that resulted in the loss of side tail fibers. In fact, the adsorption profile of one of the mutants, GP1, is indistinguishable from that of the LA-wt (see [additional file 2]).

Clearly, our results demonstrated that the reason why the LA-wt phage, though with its overwhelming advantage, was unable to reach fixation during the production-emigration experiment is because all the HA-Stf-derived phages that were able to diffuse out of the plaques are phenotypically and genotypically side-tail-fiber-less LA phages. Apparently, diffusing out of a biofilm-like matrix exerts such a strong selective pressure that only phages with reduced adsorption rates can successfully diffuse out the confine of the agar gel.

## Discussion

### Effect of the adsorption rate on the settlement rate

Since the processes of adsorbing onto host cells in liquid and settling onto biofilm are similar to each other, both governed by the diffusion of phage particles in the medium, we expected that the phage with a higher adsorption rate in liquid culture would also have a higher settlement rate on the biofilm. However, our result suggested the possibility of an optimal adsorption rate for settlement, for HA-J_1077-1 _has the highest settlement rate but an intermediate adsorption rate. As discussed above, we believe this may be an epiphenomenon. To be able to settle on the biofilm and initiate infection, a phage needs to penetrate (diffuse into) the biofilm matrix and adsorb onto the bacterial cells embedded inside it. That is, besides the adsorption rate, the virion size (morphology) may also determine the success of the settlement. In our phage collection, the phage with the highest adsorption rate (*i.e*., HA-Stf) also has the largest virion bulk, due to the presence of side tail fibers. Therefore, the seemingly reduced settlement rate for the HA-Stf phage may simply be due to its larger size, rather than its very high adsorption rate. However, to test such a hypothesis more phage strains with high adsorption rates but without the side tail fibers would be needed. Nevertheless, our results demonstrated that different genetic solutions to solve the same phenotypic imperative in one environment (*e.g*., high adsorption rate in liquid culture) could have different fitness ramifications when in a different environment.

### Effects of adsorption rate and the crowded biofilm environment

Our results showed negative relationships between adsorption rate and plaque size, plaque productivity, and emigration rate, but only to a certain extent. It seems that the adsorption rate has a diminished effect on these traits once it has exceeded certain values. Though there are several theoretical models on the development of plaque size [21,22] and references therein] and plaque fecundity (productivity) [23], unfortunately, these models do not arrive at a consensus on the effect of adsorption rate on plaque size or productivity. Interestingly, the reaction-diffusion model proposed by Yin and colleagues does show an optimal adsorption rate for plaque size (expressed as wavefront velocity) [24]. But a later study showed conflicting results, depending on how the model was analyzed [22]. Despite these inconsistent results on phage plaque size, there seems to be an opinion that low adsorption rate would result in a larger plaque size [25].

One possible reason for our observed patterns may be because of the very crowded condition of the bacterial lawn embedded in top agar gel. Because of high cell density in the top agar layer, the proportion of high-adsorption phages that are able to diffuse for a long distance before encountering, and subsequently binding to, neighboring bacterial cells would be smaller than the low-adsorption phages. As a result, more low-adsorption phages would be able to diffuse farther, thus generating a larger plaque size. Because high-adsorption phages would likely be adsorbed onto bacterial cells in their immediate surrounding, restricted local diffusion would also increase the chance of multiple infection. Even though the burst size of multiple infection is generally larger than that of the single infection, the average phage progeny produced per infecting phage would be smaller [26] (but see [27], p. 331), thus contributing to the overall reduced plaque productivity for the high-adsorption phages. Similarly, low adsorption rate means less phage progeny are bound to the host cells, thus can freely emigrate (diffuse) out of the top agar gel, resulting in a higher emigration rate.

Even though the plaque size is mainly mediated through cell density in the biofilm, we do observe several instances of significant differences between 0.27% and 0.8% top agar concentrations. Furthermore, the existence the phage-borne polysaccharide depolymerase [28,29] suggests that such enzyme is an evolutionary response to overcome the obstacle posed by the exopolysaccharide, the main scaffold of the biofilm (equivalent to the top agar gel in our study). That is, the concentration of the exopolysaccharide is also important in determining how freely the phages would be able to move within and between biofilm patches.

### Evolution of low adsorption rate and the appearance of the GP phages

The advantage of low-adsorption phage in the biofilm environment is most vividly demonstrated by its competitive rise in frequency during the production-emigration transfer cycles. In fact, the same pattern was also observed when there is no top agar layer (*i.e*., 0% top agar - see [additional file 3]). Even though we have not conducted the serial transfer experiments with the complete settlement-production-emigration cycle, the summary in Table 1 makes it apparent that low adsorption rate is advantageous when the phage fitness is dependent on its ability to be transmitted to the next locale.

The selective pressure is such that the most remarkable result from our study is the emergence of large-plaque variants (GP phages in Supplemental Table Two sup) evolved from the ancestral HA-Stf during the transfer. Apparently, the HA-Stf plaques contain a mixture of phages, possibly with high proportion of the GP phages. For example, there would be on average ~500 phages emigrated out of the HA-Stf plaque under the condition of 0.8% top agar gel (calculated as the product of the determined HA-Stf emigration rate of 1.08 × 10^-4 ^and the average HA-Stf plaque productivity of 4.60 × 10^6^). If all emigrants are GP phages (an overestimation, but most likely not by much) and would actually emigrate at a rate that is similar to the LA-wt phage (*i.e*., 9.33 × 10^-4^), it means that on average an HA-Stf plaque would contain ~5.36 × 10^5 ^GP phages (= 500/9.33 × 10^-4^), almost 12% of the total phage population in an average HA-Stf plaque (= 5.36 × 10^5^/4.60 × 10^6^). Such a high proportion of GP mutants within HA-Stf plaques could be explained by the strong selective advantage of these low adsorption rate phages in a biofilm-like environment, but also by the early appearance of these mutants during plaque formation.

Indeed, the observation that most GP mutations were the results of insertion/deletion mutations at the homonucleotide runs of the *stf *gene suggests that the appearance of the GP phages is almost inevitable. A high proportion of low adsorption rate mutants within a HA-Stf plaque may also partially explain the overall higher plaque productivity when compared to the HA-J_1077-1 _phages (see Figure 3).

The appearance of low adsorption phages in our study is reminiscent of a recent study on the effect of restricted host movement on the evolution of pathogen infectivity. By manipulating the food medium, Boots and Mealor [30] were able to restrict the movement of the host and select a low infectivity variant from the high viscosity environment, as predicted by the theoretical model [31]. On the face of it, our current results seem to confirm the prediction [31] that, under an environment where the host movement is highly restricted, a pathogen should evolve toward low infectivity (equivalent to the adsorption rate in our present work) to maximize transmission. The similar results, evolution of low infectivity and evolution of low adsorption rate, seem to suggest a similar underlying epidemiological process. Another hypothesis explaining our results is illustrated by phage populations infecting *Halobacterium cutirubrum *that naturally occurs in the Jamaican salt pond [32]. In this particular case, low adsorption wild-type isolates quickly evolved to high adsorption variants in laboratory cultures. It is hypothesized that the periodic boom-and-bust of the host population induced by fluctuation in salinity caused by rainfall would produce abundant host debris in the salt pond. The low adsorption phage isolates are selected in this environment to reduce unproductive contact with cell debris. Once in the laboratory culture, cell debris is no longer the dominant factor in reducing phage population size, thus the evolution of high adsorption rate. That is, even with global mixing in the salt pond, the low adsorption variant still prevails, a contradiction to most epidemiological predictions. We believe a similar dynamics also happens in our study, with the low adsorption phage strain committing less "suicide" inside the lysis plaque, which is believed to be full of cell debris.

### Advantage of hypermutable side tail fiber gene in the face of changing habitats

From our previous [13] and current studies we can come to a general conclusion on the evolutionary trajectory of the phage adsorption rate in two diametrically different environments: the planktonic fluid, simulated by the liquid culture in the laboratory, and the benthic biofilm, approximated by the double-layer agar plate. In reality, these two habitats would most likely appear together, with the biofilm being constantly, or intermittently, covered with flowing fluid. Since the bacterial hosts are found in both habitats, the phage would face two opposing selective pressures on the evolution of its adsorption rate. During the planktonic phase, a high adsorption rate would be beneficial, while the opposite is true during the benthic biofilm phase. In response to selection of high adsorption rate in the planktonic habitat, at least for the phage λ, two genetic solutions are possible: acquisition of the side tail fiber (represented by the HA-Stf) and improvement of the tail fiber (represented by the HA-J_1077-1_). However, these two evolutionary responses may likely have different implications when the phage is experiencing selection in the biofilm habitat.

For example, during serial transfer experiments, we have not witnessed any large-plaque variants from the HA-J_1077-1 _phage. Assuming that under our experimental condition, the large plaque phenotype is a reliable indicator for low adsorption rate, this result would suggest that it is difficult to obtain low-adsorption variants from HA-J_1077-1 _phage. However, as we have shown, the low-adsorption variants, the GP phages, are readily available from the HA-Stf phage. Furthermore, by subjecting one of the GP mutants, GP1, and its host *E. coli *in a chemostat culture, we are able to evolve the large-plaque variant back to a small-plaque version (called GP1R7 - see [additional file 2]) with an increased adsorption rate. A similar pattern of large-to-small plaque size evolution has also been observed elsewhere with λ phage [33]; though, in that case, it is not clear if the adsorption rate has also evolved correspondingly.

At this point, we do not know the relative importance of the planktonic and benthic habitats in determining the overall phage fitness, or whether the phage would evolve an optimal adsorption rate as a compromise for negotiating between these two habitats. However, it is interesting to speculate that the polymorphism of presence/absence of the side tail fibers, brought about by the high mutation rate, a consequence of various homonucleotide runs in the gene sequence, may already be a genetic solution at the population level that approximates the optimal adsorption rate, allowing the phage to negotiate between these two extreme habitats. That is, the gain/loss cycle of the side tail fiber may be a superior evolutionary route than simply evolving the tail fiber gene through sequential compensatory mutations. We also do not know the prevalence of phages carrying disposable side tail fibers or if genes encoding these disposable side tail fibers inevitably contain homonucleotide runs in their sequences. However, our experimental system, namely diffusing out of a lysis plaque, may prove to be a simple yet powerful way to screen other phages for the existence of disposable side tail fibers.

Clearly, the validity of the above discussion depends on how realistically the top agar gel simulates the real biofilm. It is to be noted that the biofilm is a much more complex and intricate structure than the simple top agar gel, presumably with cells uniformly distributed in it. The presence of various water channels and passageways [2,3] inside the biofilm suggest that the bacterial cells are not evenly distributed. How the internal structure of the biofilm affects the evolution of phage adsorption rate would be an interesting subject for further study.

## Conclusion

Our results showed that the spatially restricted and crowded biofilm is a very different environment from the liquid culture and would select for a low adsorption rate in phage population. Only phages that are endowed with disposable side tail fibers and facilitated by high mutation rate can quickly adapt to both the planktonic liquid culture and the benthic biofilm habitats. This discovery implies that disposable side tail fibers may be an adaptation to the constantly changing habitats encountered by phages and could help to explain the maintenance of the *stf *gene in phage λ's genome. Our discovery further implies that phages not endowed with disposable side tail fibers may only be successful in a certain habitat, thus becoming specialized in hosts in either the planktonic or benthic habitat.

## Methods

### Bacterial and phage strains, plasmids, and primers

Bacterial and phage strains, plasmids, and primers used in this study are listed in [additional file 4]. The bacteriophage λ is a virus with a lytic and a lysogenic lifestyle. Because we were only interested by the lytic lifestyle of λ, we used a strain carrying the *cI857 *thermosensitive mutation, preventing lysogeny when cells are grown at 37°C. Unless stated otherwise, bacteria cultures were grown in LB medium with antibiotics when appropriate. The concentrations of antibiotics were as follows: 100 μg/mL for ampicillin and 10 μg/mL for chloramphenicol.

### Construction of phage strains

The goal of phage strain construction is to construct isogenic phage strains that differ in two traits: adsorption rate and marker state. The laboratory wild-type (wt) phage λ strain is designated as the low-adsorption (LA) strain, because it lacks the side tail fiber (Stf) and has the wt tail fiber J. Two types of high-adsorption (HA) phages were constructed: (1) the HA-Stf phage, which retains the wt J but regains the Stf, and (2) the HA-J_1077-1_, which has three mutations (E1075V, A1076S, and V1077A) in its J [15]. The construction of HA-Stf phage has been described previously [13]. Similar methodology [13] was also used to construct the HA-J_1077-1 _phage. The *J*_1077-1 _allele [15] was constructed by site-directed mutagenesis using the QuickChange protocol [13], with pZE247-6 (R. Gallet, unpublished data), a derivative of pZE1-J-stf [13] containing the V1077A mutation in *J*, as the template and J1077-labFor/J1077-labRev as the primer pairs. The resulting plasmid, pZE1077-1, which contains three mutations (E1075V, A1076S, and V1077A) when compared to the wt *J*, was transformed into a λ lysogen SYP052 [13], which has a deletion encompassing part of *J*, entire *lom *and part of *orf401*. After thermal induction, only the recombinant can form plaques. To differentiate different phage strains, a functional and defective α-fragment of *E. coli*'s β-galactosidase were fused at the end of phage λ's endolysin protein R, resulting in blue or clear ("white") plaques [13]. The identities of various strain constructs were confirmed by DNA sequencing. Details of the construction have been described previously [13].

### Plating condition

One hundred μL of appropriately diluted phage solution was pre-adsorbed with 100 μL of freshly grown *E. coli *XL1 blue cells at room temperature for 20 min. The mixture was then mixed with 3 mL of molten H-top agar (10 g tryptone, 8 g NaCl, and 8, 5.3 or 2.7 g agar per liter of H_2_O) with or without the addition of ~14.3 mM IPTG and ~0.06% X-gal and poured onto a plate containing 40 mL of freshly prepared LB-agar (10 g tryptone, 5 g yeast extract, 10 g NaCl and 15 g agar per liter of H_2_O). The plate was incubated overnight at 37°C.

### Determination of the adsorption rate

Approximately 4.5 × 10^4 ^phages were inoculated in a flask containing 10 mL of TB medium (1% tryptone and 0.5% NaCl) with ~10^7 ^(for the HA-Stf strain) or ~10^8 ^(for the LA and HA-J_1077-1 _strains) cells/ml. Two different cell concentrations were used because lower cell concentration allows a more precise determination of free phage concentration for phages with a very high adsorption rate, like HA-Stf. The experiments were performed at 37°C with constant shaking (250 rpm/min) for 15 minutes. Overnight cultures of *E. coli *XL1 Blue in the stationary phase, rather than the typical exponential phase, were used. At time 0, 5 and 15 min, 300 μl of the culture was withdrawn and immediately filtered on a 0.2 μm 96-well filter plate (Pall, East Hills, NY). The number of free phages in each sample was then determined by plating. Six replicates were performed for each phage strain. An exponential function (y = *be*^-*at*^), where *a *and *b *are the parameters to be estimated, and *t *the time, was used to fit the data from individual experiments. The adsorption rate (with unit of phage^-1^cell^-1^mL^-1^min^-1^) was obtained by dividing the estimated parameter *a *(with unit of phage^-1^mL^-1^min^-1^) with each determined cell concentration.

### Determination of phage plaque productivity

To estimate the phage productivity in plaques, individual agar plugs, each containing a single plaque, were removed from the agar plate using 1 mL pipette tips that had their tip-ends enlarged by cutting with a sterilized razor blade. The resulting agar plugs were extracted with 1 mL of the TB medium and a glass tissue homogenizer with a Teflon plunger (VWR). The extractate was plated in triplicates to determine the number of phages per plaque. The productivity for each phage strain was estimated by extracting 18 random plaques (nine with the blue marker and nine with the white marker) per strain per top agar concentration.

### Determination of the phage settlement rate

To determine the effect of adsorption rate on phage's ability to settle on the cells embedded in top agar, 5 mL of TB medium containing ~5 × 10^3 ^marked LA-wt phages and ~5 × 10^3 ^marked HA-Stf or HA-J_1077-1 _phages alone were placed on an LB agar plate with 100 μl overnight culture of *E. coli *XL1 Blue cells embedded in 3 mL molten H-top agar mixed with IPTG and X-gal (see above). After 30 minutes of incubation, the solution was aspirated and the plate dried for 10 minutes before overnight incubation at 37°C. Plaques emerged in the next day were counted and treated as the number of phages able to settle from the liquid phase onto the top agar. The experiment was performed twelve times with both marker states (six times with LA-wt-blue vs. HA-Stf-white and six times with LA-wt-white vs. HA-Stf-blue) per top agar concentration. Since HA-J_1077-1 _was included in the experiment at a later time, its settlement rate was conducted alone without the presence of the LA-wt strain.

### Serial transfer of the production-emigration cycle

To determine the effect of adsorption rate on phage's ability to produce progeny and emigrate out of the current plaque, approximately 20 marked LA-wt and 180 marked HA-Stf or HA-J_1077-1 _phages were plated on XL1 Blue lawn and incubated at 37°C for overnight (see above for plating condition). The next day, 5 mL of TB medium was poured on the plate and incubated at room temperature. After 30 min, the liquid was aspirated, collected, and appropriately diluted so that approximately a total of 100 - 200 phages were plated. The extraction process was repeated three times. The emigration rate was calculated as follows:  where *ϕ*_a_^*dif *^is the number of type *a *diffused phages and *ϕ*_a_^*tot *^is the total number of *a *phages on the plate, which was estimated as the product of the average plaque productivity (*P*_*a*_), by the number of plaques on the plate (*n*_*a*_).

Besides the full-strength standard top agar concentration (0.8%), 2/3- (0.53%) and 1/3-strength (0.27%) of concentrations were also used to determine the effect of top agar concentration on phage diffusion. The experiment was performed twelve times with both marker states (six times with LA-wt-blue vs. HA-J_1077-1_-white or HA-Stf-white and six times with LA-wt-white vs. HA-J_1077-1_-blue or HA-Stf-blue) per top agar concentration.

### Determination of plaque size

Plaque size was estimated by analyzing the images of plates showing lysis plaques. The images were taken with Qcount (Spiral Biotech, Inc.). Images of four to five replicate plates, each containing ~100 plaques were taken and analyzed using the ImageJ software. The plaque size was converted from the original unit in pixel to mm^2 ^by compared it to a standardized round image with known pixel number.

### DNA-sequencing of the HA-Stf large-plaque mutants

To ensure only a single phage genome was sequenced, large-plaque variants derived from the HA-Stf phages were first made into lysogenic strains. Previously described protocol for PCR-DNA sequencing [13] was used to sequence part of the *J *and the entire *lom, stf*, and *tfa *genes. Lysogen cells, containing independent large-plaque prophage, were used as the DNA template for PCR amplification. Sequencing primers are listed in [additional file 4].

### Statistics

Phage settlement rate, plaque productivity, emigration rate, and the proportion of HA-Stf or HA-1077-1 phages in populations during the production-emigration competitive transfer experiment were analyzed with linear mixed models (*lme *function of *nlme *package, R version 2.4). In all models, replicate was used as a random factor (9 replicates for plaque productivity, 12 for settlement rate and emigration rate determination and HA-Stf or HA-1077-1 proportion during the production-emigration competitive transfer experiment). Top agar concentration (0.27%, 0.53% or 0.8%), phage strain (LA-wt, HA-Stf or HA-J_1077-1_, marker state of the phage strain (producing blue or white plaques) and transfer number (only in the analysis of HA-Stf or HA-1077-1 proportion in populations) were used as categorical fixed variables. Also, when analyzing plaque productivity, the variance function *varIdent *(library nlme) was used to accommodate heterogeneity in variance among different top agar treatments [34].

## Authors' contributions

RG: original idea, project design, λ strain constructions, experiments, statistical analyses, writing. YS: λ strain constructions. INW: project design, statistical analyses, writing, supervision. All authors read and approved the final manuscript.

## Supplementary Material

Additional file 1 **Partial *stf *sequences of independent large-plaque variants derived from the HA-Stf phage**. Tables showing partial *stf *sequences of independent large-plaque variants derived from the HA-Stf phage. Click here for file

Additional file 2 **Adsorption profiles of HA-Stf, LA-wt phages, GP1 and GP1R7**. Figure showing examples of HA-Stf, LA-wt phages, GP1 and GP1R7 adsorption profiles. Click here for file

Additional file 3 **Competitive serial transfer experiments between LA-wt and HA-Stf**. Figure showing the results of competitive serial transfer experiments between LA-wt and HA-Stf in 0%, 0.27%, 0.53% and 0.8% agar. Click here for file

Additional file 4 **List of bacterial and phage strains, plasmids, and primers**. Tables showing all bacterial and phage strains, plasmids, and primers used in this study Click here for file
