# Supplementary material for: High adsorption rate is detrimental to bacteriophage fitness in a biofilm-like environment
Source: BMC Evol Biol. 2009 Oct 5;9:241. doi: 10.1186/1471-2148-9-241 (PMC2762979; doi:10.1186/1471-2148-9-241)
Supplement: Additional file 1 — Partial stf sequences of independent large-plaque variants derived from the HA-Stf phage. Tables showing partial stf sequences of independent large-plaque variants derived from the HA-Stf phage. [file 1471-2148-9-241-S1.DOC]

# Partial *stf* sequences of independent large-plaque variants derived from the HA-Stf phage.

| Strain | Mutation | Sequence | Position |
| --- | --- | --- | --- |
| λ*PaPa* | C deletion | GAAAAGCC:CACTGGAC | 20835 |
| GP1 | G deletion | ACACGGGG:CTCATGCT | 21572 |
| GP2 | A insertion | ATTCTGGC**A**AAAAAATT | 21181 |
| GP3 | T to C substitution | CTGCACCA**C**TCAGCTGA | 19714 |
| GP4 | G deletion | ACACGGGG:CTCATGCT | 21572 |
| GP5 | A deletion | CGGAAAAA:GTGCCGCA | 20205 |
| GP6 | G insertion | CACGGGGG**G**CTCATGCT | 21573 |
| GP7 | T to C substitution | CGGTGGGC**C**CAGAGAAT | 19767 |
| GP8 | G deletion | ACACGGGG:CTCATGCT | 21572 |
| GP9 | G insertion | CACGGGGG**G**CTCATGCT | 21573 |
| GP10 | G insertion | AACAGGGG**G**CCGCGGGT | 21606 |
| GP11 | G insertion | CGGCGGGG**G**AGTGCGTC | 21562 |
| GP12 | C deletion | TCCCGTGG:CATCAGAT | 21263 |
| GP13 | ?*^a^* |  |  |
| GP14 | G insertion | CGGCGGGG**G**AGTGCGTC | 20562 |
| GP15 | C to T substitution | TTCACAAC**T**GGGGACGC | 19897 |
| GP16 | T to C substitution | TACAGAAC**C**GCACCATT | 19707 |
| GP17 | A insertion | GCAAAAAA**A**TTCGGCAA | 20666 |
| GP18 | ?*^a^* |  |  |
| GP19 | A deletion | GGACGAAA:CCACATCG | 21494 |
| GP20 | T deletion | GGCTGGAG:CAGTATGG | 21661 |
| GP22 | G deletion | ACACGGGG:CTCATGCT | 21572 |

*^a^* No mutation was found in the *J-lom-stf-tfa* region.
