# Supplementary material for: High adsorption rate is detrimental to bacteriophage fitness in a biofilm-like environment
Source: BMC Evol Biol. 2009 Oct 5;9:241. doi: 10.1186/1471-2148-9-241 (PMC2762979; doi:10.1186/1471-2148-9-241)
Supplement: Additional file 2 — Adsorption profiles of HA-Stf, LA-wt phages, GP1 and GP1R7. Figure showing examples of HA-Stf, LA-wt phages, GP1 and GP1R7 adsorption profiles. [file 1471-2148-9-241-S2.DOC]

# Adsorption profiles of HA-Stf, LA-wt phages, GP1 and GP1R7

Examples of adsorption profiles of HA-Stf (blue square), LA-wt phages (blue circle), GP1 (red triangle) and GP1R7 (orange diamond). GP1 is a HA-Stf mutant making large plaques after one transfer in the production-emigration competitive experiment. GP1R7 is a GP1 revertant making small plaques and obtained after a three-day culture in chemostat with *E. coli* XL1blue. The proportion of free phages remaining in solution is plotted against time. For all phages ~10^8^ cells/mL of stationary *E. coli* XL1 Blue cells were used.
