# Supplementary material for: High adsorption rate is detrimental to bacteriophage fitness in a biofilm-like environment
Source: BMC Evol Biol. 2009 Oct 5;9:241. doi: 10.1186/1471-2148-9-241 (PMC2762979; doi:10.1186/1471-2148-9-241)
Supplement: Additional file 3 — Competitive serial transfer experiments between LA-wt and HA-Stf. Figure showing the results of competitive serial transfer experiments between LA-wt and HA-Stf in 0%, 0.27%, 0.53% and 0.8% agar. [file 1471-2148-9-241-S3.DOC]

# Competitive serial transfer experiments between LA-wt and HA-Stf.

Curves represent the proportion of LA-wt in the carrier liquid medium as a function of transfer number. The experiments were performed at 0 (purple triangle), 0.27 (blue diamond), 0.53 (green circle) and 0.8 % (red star) top agar concentrations. Error bars represent 95% confidence intervals.
