# Supplementary material for: High adsorption rate is detrimental to bacteriophage fitness in a biofilm-like environment
Source: BMC Evol Biol. 2009 Oct 5;9:241. doi: 10.1186/1471-2148-9-241 (PMC2762979; doi:10.1186/1471-2148-9-241)
Supplement: Additional file 4 — List of bacterial and phage strains, plasmids, and primers. Tables showing all bacterial and phage strains, plasmids, and primers used in this study [file 1471-2148-9-241-S4.DOC]

# List of bacterial and phage strains, plasmids, and primers.

| Name | Relevant genotype | Reference |
| --- | --- | --- |
| Bacterial strain | | |
| XL1 Blue | *E. coli* K12, *lacZ*ΔM15 | Stratagene |
| RG31 | MG1655(λ*cI857 Swt R::LacZα^+^ stf^-^ J_1077-1_*) | This study |
| RG61 | MG1655(λ*cI857 Swt R::LacZα^-^ stf^-^ J_1077-1_*) | This study |
| SYP045 | MC4100(λ*cI857 Swt R::LacZα^+^ stf^-^ J^+^*) | [13] |
| SYP046 | MC4100(λ*cI857 Swt R::LacZα^+^ stf^+^ J^+^*) | [13] |
| SYP049 | MC4100(λ*cI857 Swt R::LacZα^-^ stf^-^ J^+^*) | [13] |
| SYP056 | MC4100(λ*cI857 Swt R::LacZα^-^ stf^+^ J^+^*) | [13] |
| SYP052 | MC4100(λ*cI857* *Swt R* Δ(*J-orf401*)::*Cam*) | [13] |
| Phage strain | | |
| All phage strains were obtained by thermally-inducing the above lysogen strains [13]. | | |
| Plasmid | | |
| pZE1-J-stf | Contains the genomic region of λ encompassing part of *J*, entire *lom*, *orf401*, and part of *orf314* (*orf401* and *orf314* are the two part of the *stf* gene when a frameshift is inserted in the gene) | [13] |
| pZE1077-1 | Contains the genomic region of λ encompassing part of *J* (containing the three 1077-1 mutations - E1075V, A1076S, and V1077A), entire *lom*, *orf401*, and part of *orf314* | This study |
| Primers – strain construction | | |
| J247-6_For | GGCGAACGAGGCGGCACAGGTGTTCTCCCG | This study |
| J247-6_Rev | CGGGAGAACACCTGTGCCGCCTCGTTCGCC | This study |
| J1077-1ab_For | GATGGCGCGGCGAACGTGTCGGCACAGGTGTTCTCC | This study |
| J1077-1ab_Rev | GGAGAACACCTGTGCCGACACGTTCGCCGCGCCATC | This study |
| Primers – DNA sequencing | | |
| JtoStf_For | GCGCGACGTCAGGTTGAAACCAGCACGCG | [13] |
| J2942_For | CTGTACGATAAACGGTACGC | This study |
| J18656_Rev | CACATCGAATACGTTGTCC | This study |
| Lom-Stf19567_For | CTGACGGATTCATCGTTGGG | This study |
| Stf20508_Rev | GAGGCGCTCCGTTCCGCTGC | This study |
| JtoStf_Rev | GCGCTCTAGACAGCACGACCGCTGGCGGG | [13] |
| Stf21501_Rev | CGTCCCGTAATCAAACGACG | This study |
| Stf20064_For | GATGCCAGTGCATCAGCTG | This study |
| Stf21246_For | AATTCGGCCTTTCCGGCAGG | This study |
| Stf 20364_For | GCGGTGGCCTCAAAAGAGGC | This study |
| Tfa_For | CGTCCTGATGCAGGGGCAGGCG | This study |
| Tfa22214_Rev | AATAAATAACGCGTCGCCGG | This study |
| Tfa-EA47 22645_Rev | CCTGAAGATAATAATCGCC | This study |
